# Infectious Disease Underreporting Is Predicted by Country-Level Preparedness, Politics, and Pathogen Severity

**DOI:** 10.1089/hs.2021.0197

**Published:** 2022-08-11

**Authors:** Amanda J. Meadows, Ben Oppenheim, Jaclyn Guerrero, Benjamin Ash, Rinette Badker, Cathine K. Lam, Chris Pardee, Christopher Ngoon, Patrick T. Savage, Vikram Sridharan, Nita K. Madhav, Nicole Stephenson

**Affiliations:** Amanda J. Meadows, PhD, is a Data Scientist/Modeler, Metabiota, San Francisco, CA.; Ben Oppenheim, PhD, MA, MSc, is Vice President of Product, Policy, and Partnerships, Metabiota, San Francisco, CA.; Jaclyn Guerrero, MPH, is an Advisor, Epidemiology Products, Metabiota, San Francisco, CA.; Benjamin Ash, MS, is Manager of NRT Data, Metabiota, San Francisco, CA.; Rinette Badker, MSc, is a Senior Epidemic Analyst, Metabiota, San Francisco, CA.; Cathine K. Lam, ACAS, is a Data Scientist/Actuary, Metabiota, San Francisco, CA.; Chris Pardee, MS, is Senior Manager of Data Acquisition, Metabiota, San Francisco, CA.; Christopher Ngoon, MS, is a Senior Data Analyst, Metabiota, San Francisco, CA.; Patrick T. Savage is a Data Quality Analyst, Metabiota, San Francisco, CA.; Vikram Sridharan, MS, is a Senior Data Scientist and Technical Product Manager, Metabiota, San Francisco, CA.; Nita K. Madhav, MSPH, is Chief Executive Officer, Metabiota, San Francisco, CA.; Nicole Stephenson, DVM, MPVM, PhD, is Senior Director of Data Science and Modeling, Metabiota, San Francisco, CA.

**Keywords:** Underreporting, Reporting bias, Infectious disease, Public health preparedness/response

## Abstract

Underreporting of infectious diseases is a pervasive challenge in public health that has emerged as a central issue in characterizing the dynamics of the COVID-19 pandemic. Infectious diseases are underreported for a range of reasons, including mild or asymptomatic infections, weak public health infrastructure, and government censorship. In this study, we investigated factors associated with cross-country and cross-pathogen variation in reporting. We performed a literature search to collect estimates of empirical reporting rates, calculated as the number of cases reported divided by the estimated number of true cases. This literature search yielded a dataset of reporting rates for 32 pathogens, representing 52 countries. We combined epidemiological and social science theory to identify factors specific to pathogens, country health systems, and politics that could influence empirical reporting rates. We performed generalized linear regression to test the relationship between the pathogen- and country-specific factors that we hypothesized could influence reporting rates, and the reporting rate estimates that we collected in our literature search. Pathogen- and country-specific factors were predictive of reporting rates. Deadlier pathogens and sexually transmitted diseases were more likely to be reported. Country epidemic preparedness was positively associated with reporting completeness, while countries with high levels of media bias in favor of incumbent governments were less likely to report infectious disease cases. Underreporting is a complex phenomenon that is driven by factors specific to pathogens, country health systems, and politics. In this study, we identified specific and measurable components of these broader factors that influence pathogen- and country-specific reporting rates and used model selection techniques to build a model that can guide efforts to diagnose, characterize, and reduce underreporting. Furthermore, this model can characterize uncertainty and correct for bias in reported infectious disease statistics, particularly when outbreak-specific empirical estimates of underreporting are unavailable. More precise estimates can inform control policies and improve the accuracy of infectious disease models.

## Introduction

Infectious disease surveillance systems and accurate disease reporting are essential to identify public health policy priorities, make data-driven decisions regarding disease mitigation, and inform statistical models of disease progression for forecasting and other analytic purposes.^1-3^ However, many diseases are underreported due to under-ascertainment, misdiagnosis, inadequate public health and disease surveillance infrastructure, or failure to comply with disease reporting requirements.^1^ A variety of analytical methods are often applied to construct a more accurate estimate of true disease incidence. Examples of these methods include capture-recapture studies, inference from seroprevalence studies, and projections from models.^4^ Although such analytical methods provide valuable insights about infectious disease reporting rates, many approaches are time-consuming, require large volumes of data (including, in some cases, the collection of new primary data), and may only characterize reporting completeness for a specific population and disease of interest.

Previous research has shown that disease-specific factors such as symptom severity, perceived seriousness, social stigma, and mortality rates influence reporting rates.^3,5^ For example, pathogens causing severe disease or death are more likely to be reported than those with less serious symptoms, in part because individuals infected are more likely to seek healthcare.^6^ Diseases of specific public health interest, such as sexually transmitted infections (STIs), are also more likely to be reported because specific programs are in place to prevent and track these pathogens.^3^

Reporting rates for the same disease can vary spatially, with country-specific economic, institutional, and political factors affecting national reporting rates.^7^ Oppenheim et al^8^ developed and published an Epidemic Preparedness Index (EPI) that summarizes national capacity to detect and respond to infectious disease outbreaks based on a holistic set of capabilities. Countries with higher epidemic preparedness were found to report infectious disease outbreaks more quickly; the analysis of EPI scores did not, however, examine the relationship between preparedness and reporting rates. While the EPI serves as a proxy for national capacity to detect and report an infectious disease epidemic, it does not measure political will to do so. Countries may have strong incentives to underreport infections, for example to avoid potential political, social, or economic instability,^9^ which could result from news of a widespread or potentially deadly pathogen, or to avoid potential reputational damage with domestic and international audiences from failing to effectively manage a disease outbreak.^10,11^

The importance of accuracy in global disease reporting has never been more relevant given the ongoing COVID-19 pandemic. Identifying factors associated with underreporting can provide a data-driven starting point on how to improve infectious disease reporting, help epidemiological modelers build more reliable disease spread models, and guide public health response to outbreaks. In our study, we combined epidemiological and social science theory to identify factors that may influence empirical reporting rates and used model selection techniques to build a model that predicts pathogen- and country-specific reporting rates. Pathogen-specific factors we considered include case fatality rate and sexual transmission. Country-specific factors include political variables from the Varieties of Democracy dataset^9^ measuring public sector corruption, government censorship, media bias, regime type, as well as epidemic preparedness (measured by the EPI) and national income (measured categorically by the World Bank country income group).

## Methods

### Reporting Rate Literature Search

We conducted a literature search to identify studies that provide data on infectious disease reporting rates specific to a country or a pathogen. Studies were considered relevant if they reported multiplication or expansion factors, estimated reporting rates, or if they described a method to correct official case incidence data. Studies giving an anecdotal range of underreporting without providing methodology or a source to support the claim were excluded. We considered 84 pathogens that ranged in symptom severity, place of occurrence, prevalence, transmission mode, and mortality rate.^12^ We searched for pertinent studies via Google Scholar and PubMed using the terms: “underreporting model,” “infectious disease underreporting,” “infectious disease reporting bias,” and “<pathogen> AND underreporting.” Pathogens entered in the “<pathogen> AND underreporting” search are listed in Supplemental Table (www.liebertpub.com/doi/suppl/10.1089/hs.2021.0197). Our inclusion criteria for studies were: (1) the study must provide a quantitative estimate of reporting completeness, (2) the study must estimate reporting rates at the national level or subnational spatial resolution, and (3) the study must describe reporting rates for a specific, named pathogen. Studies that estimated reporting rates for diseases or clinical manifestations attributed to multiple pathogens (eg, encephalitis) were excluded unless the study specified a single causative agent. Studies that estimated reporting rates at geographic resolutions less granular than the country level, such as regional or continental (eg, the European Union), were excluded, so that the response variable would align with country-specific predictor variables. Studies that estimated reporting rates at a more granular resolution than country level (eg, state or province) were included and assigned to the national level.

The Abstract, Method, and Results sections of the returned papers were reviewed to determine if the study provided a quantitative estimate of reporting completeness. Additional sources were identified from the references and cited literature of the returned studies. The literature search was completed during July 2019. We recorded reporting rate estimates found in each study, usually expressed as a proportion between 0 and 1, but there is a possibility that reporting rates could exceed 1 if a disease was overreported. If a study estimated reporting rates over a period spanning more than a year, we recorded the first year as the year associated with the study (eg, a study spanning 2001-2002 would be coded as 2001). Multiple reporting estimates were sometimes available from the same study, and these were recorded as independent data points if the study gave independent reporting estimates for multiple pathogens or over multiple years. Additionally, there were studies where different sources independently estimated reporting completeness for the same pathogen over similar time scales for the same country. In such cases, we condensed the dataset to 1 data point per pathogen/country/year combination by taking the mean of reporting rate estimates from the different sources (outcomes were robust to this decision).

Although our central aim was to identify country- and pathogen-specific factors that predict reporting rates, collecting reporting rates from scientific literature compiled over decades and through several different analytical methods could introduce additional patterns in the data that are not explained by pathogen- or country-specific variables. Therefore, we collected study-specific metadata that we could use diagnostically.

### Candidate Predictor Variables

#### Study-Related Variables

In addition to extracting pathogen- and country-specific reporting estimates from studies identified in our literature review, we recorded study characteristics to test if reporting estimates were sensitive to certain aspects of the study. Information extracted from these studies to be assessed as potential covariates included the year(s) of the study and whether the cases occurred during an epidemic.

#### Country-Specific Variables

There are many variables that could influence a country's capacity to accurately report infectious disease cases. However, many factors—including aspects of health system capacity and performance such as doctors, hospital beds, and health spending per capita—are likely to be correlated, which could bias model estimates. We used the EPI as a single predictor variable to summarize country-level capacity for public health surveillance and reporting.^8^ The EPI consists of 5 subindices measuring each country's economic resources, public health communications, infrastructure, public health systems, and institutional capacity (see Oppenheim et al^8^ for further index details). Countries are scored from 0 to 100, where 100 represents the highest level of preparedness and 0 represents the lowest. The EPI has been found to correlate highly (correlation coefficient of approximately 0.85) with Joint External Evaluation scores,^8^ but the EPI has much broader geographic and temporal coverage, since only a subset of countries have completed the Joint External Evaluation process. We hypothesized that countries with higher EPI scores would, on average, have higher reporting rates.

Although EPI measures national capacity to detect and respond to disease outbreaks, it does not measure a country's willingness to report disease cases. Theoretical models and empirical evidence suggest that governments that fear political opposition will tend to suppress or delay the release of potentially damaging information that could signal government weakness or incompetence.^13,14^ We hypothesized that regimes that fear political opposition would be more likely to suppress disease reporting, even if the country has the resources and health system capacity to respond to disease outbreaks. To test this hypothesis, we assessed several predictor variables from the Varieties of Democracy Project^9^ measuring government censorship, control over the media, and media bias; we expect that countries with higher levels of censorship and media bias in favor of the incumbent regime will be less likely to accurately report disease incidence data (see the Supplemental Analyses: Variable Profiles, www.liebertpub.com/doi/suppl/10.1089/hs.2021.0197, or [9] for more information on these variables). Varieties of Democracy provides time-series data that reflect historical changes as precisely as possible, so we chose the variable value corresponding to the study year. For example, if a hypothetical study estimated reporting rates for Ebola virus disease in Guinea in 2015, and a different study estimated reporting rates for measles in Guinea in 1998, the first study would be matched with the Varieties of Democracy score for Guinea in 2015, and the second would be matched with the score for Guinea in 1998.

#### Pathogen-Specific Variables

We hypothesized that more severe diseases would be associated with higher reporting rates, owing to greater rates of ascertainment and motivation to mitigate deadlier pathogens. Although disease severity can be conceptualized and measured in many ways, we included the case fatality rate (CFR; as percentage of cases resulting in death) as a measurement of disease severity in our model. We do so because CFR is a widely accepted measure, is generally correlated with other dimensions of severity such as morbidity, and is easily calculated. We obtained estimates of pathogen-specific CFR estimates from a literature search with the terms “<pathogen> AND case fatality.” If multiple estimates of CFR were identified, we selected the estimate that represented an overall measure of CFR with available treatment, rather than the CFR estimated for a specific subset of cases, such as hospitalized or untreated cases. When deciding which case fatality ratio estimate to collect, we prioritized estimates in the following order: treated CFR, unspecified CFR, untreated CFR, and hospitalized CFR. These were prioritized due to perceptions of risk rather than overall burden of mortality.

Some groups of pathogens might not directly follow the relationship between severity and reporting completeness if there are specific programs in place aimed at identifying and reporting cases of infection. STIs caused by pathogens such as *Neisseria gonorrhoeae* and *Chlamydia trachomatis* typically have low case fatality rates, but many countries have screening and contact tracing programs in place to identify, treat, and report these diseases.^3^ Therefore, we also included a binary variable coding if the pathogen causes an STI or not.

### Model Selection and Fitting

We performed univariate fractional regression using the binomial glm() function with a logit link to analyze the effect of each potential predictor variable on the proportion of cases reported before considering the variable in the global multivariable fractional regression model. We found that our results are robust to the use of alternative estimators, including beta and ordinary least squares regression. Predictor variables showing *P* < .1 were assessed in our final model after being assessed for collinearity. We tested the parsimony of the final model by using the dredge() function in the MuMIn R package version 1.46.0^15^ to perform best subsets selection assessed by Akaike information criterion corrected for sample size (AICc). The dataset used to fit our model is highly varied, which could bias the results of the model. To reduce bias in our final model, we refit the model using robust estimation, which is less sensitive to violations of the assumptions of classical regression (eg, outliers, homoscedasticity). We used the function glmrob() from the robustbase R package version 0.95-0^16^ to employ the Bianco and Yohai estimator for logistic regression. Coefficients from the fitted model were exponentiated to calculate odds ratios. To assess the importance of each variable in explaining reporting rates, we examined the sequential decrease in deviance as variables were entered in the model. We refit the model, alternating which term was entered last, and compared the decrease in deviance when each term was entered last. All analyses were performed in R version 20.6.0 (R Core Team, Vienna, Austria). All code used to perform these analyses can be accessed on our GitHub repository (https://github.com/metabiota-ameadows/underreporting).

## Results

### Reporting Rate Literature Search

A total of 42 studies published between 1990 and 2018 were selected for inclusion, yielding 255 estimates of country- and pathogen-specific reporting completeness. The studies used several different analytical methods to estimate reporting rates, such as capture-recapture, mathematical models, and serosurveys. The official reporting source used as a benchmark for reporting rates also varied by study, but examples of reference reporting sources included the World Health Organization, country ministries of health, and local public health departments. All data recorded from the literature search are also available on the manuscript GitHub repository (https://github.com/metabiota-ameadows/underreporting/tree/master/data).

Although we collected 255 estimates of reporting completeness, we included only 112 of these observations in our analysis. We excluded all observations collected for *Salmonella* and *Campylobacter* pathogens because they comprised over half of the data points collected (*Salmonella*, n = 78; *Campylobacter*, n = 52) and all the data points were from high-income, wealthy, democratic countries, mostly within the European Union (see Supplemental Analyses: GI Data Exploration). Our aim was to build a model that could be generalized to a variety of pathogens and countries; including a high proportion of data points from these 2 pathogens could bias our model toward gastrointestinal pathogens from high-income nations. The next most represented pathogens in our dataset were dengue virus (n = 39) and Ebola (n = 11); however, over 75% of the pathogens in our dataset had fewer than 5 data points. After excluding *Salmonella* and *Campylobacter*, our dataset represented 33 different years (ranging from 1971 to 2016), 36 pathogens, and 32 countries.

### Model Selection

**Figure 1.**
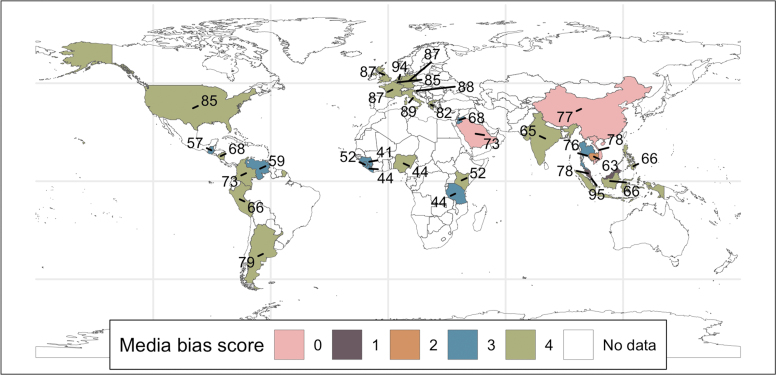
A global map showing the countries represented in the reporting rate data collected in the literature search. Countries are colored according to their Varieties of Democracy media bias score (0 = most biased; 4 = least biased) and labeled with their Epidemic Preparedness Index score (0 = least prepared; 100 = most prepared).

We performed univariate regression (Table 1) on each potential predictor variable to assess if it would be considered in model selection. In Supplemental Analyses: Variable Profiles, we provide additional detail on each variable, plot the relationship of each variable with the reporting rates collected in our literature search, and show the distribution of the variable in the dataset. None of the study-related variables (ie, study year and epidemic status) passed the univariate criterion, and therefore did not advance in the model selection pipeline. After assessing collinearity (Supplemental Analyses: Collinearity Assessment), we considered CFR, STI, epidemic preparedness, and media bias in the next stage of model selection. All these remained in the final model after checking for parsimony (Supplemental Analyses: Model Selection). Media bias is an ordinal variable ranging from 0 to 4, where 0 indicates no media coverage of opposition parties or candidates and increasing values are associated with more politically balanced media reporting. Although the majority of our data come from countries with low levels of media bias and high epidemic preparedness, countries representing all levels of media bias and epidemic preparedness are present in our data (Figure 1).

**Table 1.** Univariate Regression Performed on Each Potential Predictor Variable

Continuous Variables				
Univariate Model		Estimate	*P* Value	*n*
Public sector corruption		0.615	.008	112
CFR		0.038	.009	112
EPI		0.044	.018	112
Gov dissemination of false info (abroad)		0.704	.022	112
Gov internet filtering in practice		0.569	.034	87
Gov media censorship effort		0.438	.035	112
Media bias against opposition parties		0.434	.057	112
Print/broadcast media criticize gov		0.502	.099	112
Gov dissemination of false info (domestic)		0.431	.102	87
Study year		-0.028	.162	112

Categorical Variables				
Univariate Model	Model Term	Estimate	*P* Value	*n*
STI	Intercept (No)	-0.829	< .001	99
	Yes	1.6788	< .001	13
Income group	Intercept (Low)	-0.406	.599	4
	Lower middle	-1.785	.073	27
	Upper middle	-0.855	.377	16
	High	0.296	.716	65
Study took place during an epidemic	No	-0.564	.011	87
	Yes	-0.256	.599	25

Note: Results of univariate fractional regression models used to determine variable eligibility in the full model along with sample sizes in each group. Factors or numerical variables with *P* < .1 were assessed for collinearity before being entered into a multivariate fractional regression model. See Supplemental Analyses: Variable Profiles for Variable Definitions.

Abbreviations: CFR, case fatality rate; EPI, Epidemic Preparedness Index; STI, sexually transmitted infection.

### Final Model Results

In order to interpret the substantive impact of coefficient changes on reporting rate, it is helpful to conceptualize the reporting rate as a series of individual cases with a binary outcome, either nonreported or reported. The proportion of successful outcomes (reported) out of N true cases would be the reporting rate. All variables included in our final model had a significant effect on reporting rates, as assessed by a *P* < .05 threshold.

**Figure 2.**
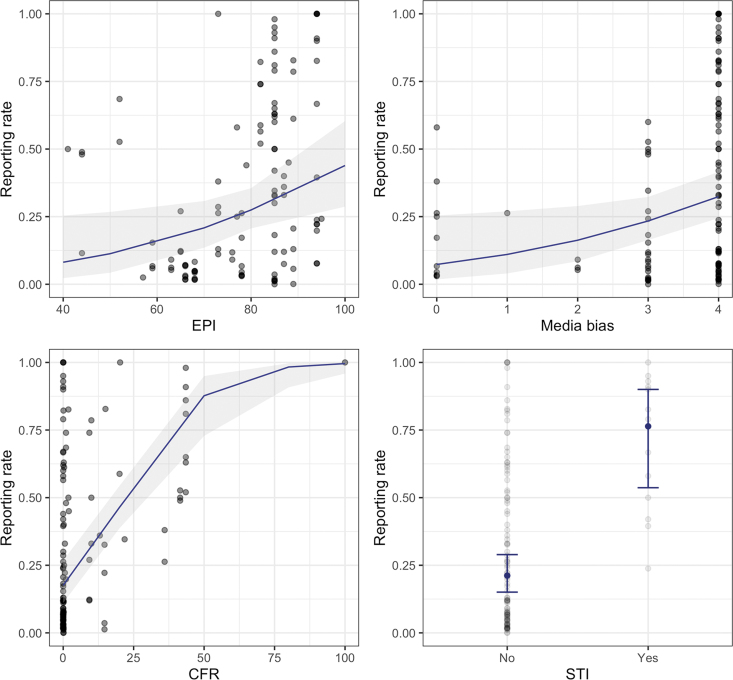
The relationship between reporting rates and Epidemic Preparedness Index score, media bias score, case fatality rate, and sexually transmitted infection grouping once the effect of other variables in the model are accounted for (navy lines in a-c; navy dots in d) and 95% confidence intervals (shaded region in a-c; error bars in d). Abbreviation: STI, sexually transmitted infection.

CFR had the largest impact on the likelihood to report (Supplemental Analyses: Model Results); cases were approximately 7% more likely to be reported for every 1% increase in CFR (Figure 2; Table 2). The model showed that STIs were approximately 12 times more likely to be reported than other pathogens. As for country-specific variables, we found that EPI scores had a positive relationship with reporting rates, in which better-prepared countries typically had higher reporting rates, as expected. For example, a country ranking 10 points higher in the EPI would be, on average, 48% more likely to report a case. Finally, we found that reporting rates were inversely associated with media bias. Moving 1 unit up the ordinal scale of the media bias indicator (meaning the country has less media bias) was associated with a 57% increase in likelihood to report a case.

**Table 2.** Results of the Fitted Reporting Rate Fractional Regression Model

Term	Description	Estimate	SE	OR	*P* Value
Intercept	Regression model intercept value	-6.198	1.510	–	<.001
STI	Is the main mode of transmission sexual? (yes/no)	2.487	0.566	12.030	<.001
CFR	Percentage of cases that result in death (0-100)	0.070	0.014	1.073	<.001
EPI	Index scoring national capacity to respond to infectious disease outbreaks (1-100)	0.036	0.016	1.040	.021
Media bias	Ordinal score indicating the balance of media reporting among political parties (0-4)	0.451	0.200	1.570	.023

Abbreviations: CFR, case fatality rate; EPI, Epidemic Preparedness Index; OR, odds ratio; SE, standard error; STI, sexually transmitted infection.

## Discussion

Underreporting compromises evidence-based infectious disease prevention and mitigation strategies, and contributes to bias in mathematical models. Such bias can be particularly problematic and important to address during ongoing epidemics and pandemics, as case counts and mathematical models often directly inform policy and disease control measures.

To the best of our knowledge, this is the first study to simultaneously consider country and pathogen characteristics as predictors of reporting rates. We find that both were significantly predictive (Table 2), although pathogen characteristics had a greater influence than country characteristics in the model (Supplemental Analyses: Model Results). Country preparedness was positively associated with reporting; this finding accords with evidence from Undurraga et al,^2^ who showed that the reporting rates of dengue episodes in Southeast Asia and the Americas were positively associated with the Health Quality Index of the country.^2^ We also found that countries with higher levels of media bias in favor of the incumbent regime were associated with a decreased likelihood of reporting infectious disease cases; this finding is consistent with case evidence from Turkmenistan by Rechel and McKee,^17^ who showed concerted efforts by political leadership to suppress infectious disease diagnosis and reporting to project an image of sound, effective governance. The pathogen mortality rate had the largest effect on reporting rates, with deadlier pathogens being more likely to be reported. Although sexually transmitted pathogens typically have low mortality rates, our model showed they were more likely to be reported than other pathogens, controlling for the mortality rate. This may be due to the fact that, although not fatal, STIs can cause serious morbidity and infertility.^18^ Additionally, many countries have specific funding and programs designed for STI surveillance to make available the resources needed for testing and reporting.^3^

Although this model furthers our understanding of factors that influence infectious disease reporting rates, there are limitations to this study. One limitation is that there are gaps in the dataset we used to fit our model, with 56% of reporting estimates collected from our literature search originating in countries scoring highest in preparedness (EPI scores ≥80). Our dataset contained reporting rates for 32 pathogens, but reporting rates were available in only 4 or fewer countries for most pathogens. Although we did not find a significant temporal trend in underreporting in the model (*P* = .16; Supplemental Analyses: Variable Profiles), there are likely temporal changes that could not be appreciated with the current dataset. Country-level variables also have likely changed over time; however, EPI scores were only available from 2016 onward, and prior values were not available to match against some reporting rate studies. As such, we used the current EPI score. Additionally, other factors likely influence reporting rates that we were unable to account for in our model. Examples of other factors that may influence reporting, but are not accounted for here, could include under-ascertainment as a result of refusal to seek healthcare for financial, religious, or cultural reasons. Additionally, another contemporary crisis or disaster may have influenced resources for reporting during the study period. Country-level variables were used, as the variables of interest are not available at a more granular level; however, there is likely substantial heterogeneity within the underlying populations that is unable to be accounted for in this analysis. Additional studies should further investigate the relationships found here to evaluate their application to scales of finer geographic resolution. Similarly, there is evidence that country preparedness can vary substantially by pathogen, as well as subnationally—some countries and subregions have stronger capacity to detect, report, and respond to particular pathogens, perhaps based on patterns of prior exposure and capacity strengthening.^19^ The EPI metric we use in this analysis does not capture these subnational and pathogen-specific dynamics; additional metric development could enable more fine-grained analyses of the relationship between preparedness, pathogen, political will, and reporting completeness.

## Conclusion

The model we describe in this article demonstrates that infectious disease underreporting is associated with various factors specific to pathogens, country health systems, and politics, and it identifies specific and measurable components of these broader factors that can guide efforts to reduce underreporting. The model can be used to extrapolate beyond the data available from observational studies to provide estimates of reporting completeness based on easily obtainable input data. Although methods such as community-based surveys, capture-recapture studies, and other analytical methods may provide the most accurate representation of infectious disease reporting completeness, our model can provide a guide to estimate the true burden of disease in the absence of time and resources to carry out more detailed studies of reporting accuracy.

## Supplementary Material

Supplemental data

Supplemental data

## Notes

[Supplementary-material SD1]

[Supplementary-material SD2]
