# Supplementary material for: Infectious Disease Underreporting Is Predicted by Country-Level Preparedness, Politics, and Pathogen Severity
Source: Health Secur. 2022 Aug 11;20(4):331–8. doi: 10.1089/hs.2021.0197 (PMC10818036; doi:10.1089/hs.2021.0197)
Supplement: Supplemental data [file Supp_TableS1.docx]

SUPPLEMENTARY INFORMATION

| Adenovirus | Hepatitis B virus | Neisseria meningitidis |
| --- | --- | --- |
| Alkhumra virus | Hepatitis C virus | New World Hantavirus |
| Bacillus anthracis (cutaneous) | Hepatitis E virus | Nipah virus |
| Bacillus anthracis (gastrointestinal) | Human Herpesvirus | Norovirus |
| Bacillus anthracis (inhalation) | Human Immunodeficiency Virus | Old World Hantavirus (Hantaan virus) |
| Borrelia burgdorferi | Human T-Lymphotropic virus | Old World Hantavirus (Puumala virus) |
| Burkholderia pseudomallei | Human herpesvirus 4 (Epstein Barr) | Omsk hemorrhagic fever virus |
| Carbapenem-resistant Enterobacteriaceae (CRE) | Human papillomavirus | Oropouche virus |
| Chandipura virus | Human respiratory syncytial virus | Pan-Resistant Carbapenem-resistant Enterobacteriaceae (CRE) |
| Chapare virus | Influenza A virus | Parvovirus B19 virus |
| Chikungunya virus | Japanese encephalitis virus | Picobirnavirus |
| Chlamydia trachomatis | Junin | Poliovirus |
| Clostridium difficile | Kyasanur forest disease virus | Rabies virus |
| Coronavirus OC43 | LaCrosse virus | Rickettsia prowazekii (Louse-borne typhus) |
| Cowpox virus | Lassa virus | Rift Valley fever virus |
| Coxiella burnetii | Leishmania (cutaneous) | Rotavirus A |
| Coxsackievirus B | Leishmania (visceral) | Rubella virus |
| Crimean-Congo hemorrhagic fever virus | Lujo virus | SARS Coronavirus |
| Dengue virus | Lymphocytic choriomeningitis virus | St. Louis encephalitis virus |
| Drug-Resistant Shigella | MERS Coronavirus | Tick-borne encephalitis |
| Drug-Resistant Streptococcus Pneumoniae | Machupo virus | Treponema pallidum |
| Eastern equine encephalitis virus | Marburg virus | Varicella-zoster virus |
| Far Eastern Tick Borne Encephalitis | Measles virus | Variola major virus (Smallpox virus) |
| Francisella tularensis | Methicillin-Resistant Staphylococcus Aureus | Variola minor virus (Alastrim) |
| Guanarito mammarenavirus | Monkeypox virus | Venezuelan equine encephalitis virus |
| Heartland virus | Mumps virus | Vibrio cholerae |
| Hendra virus | Mycobacterium tuberculosis | West Nile Virus |
| Hepatitis A virus | Neisseria gonorrhoeae | Zaire ebolavirus |

Table S1. Pathogens used as search terms in the reporting rate literature search.
