# Supplementary material for: Infectious Disease Underreporting Is Predicted by Country-Level Preparedness, Politics, and Pathogen Severity
Source: Health Secur. 2022 Aug 11;20(4):331–8. doi: 10.1089/hs.2021.0197 (PMC10818036; doi:10.1089/hs.2021.0197)

Supplementary Analysis: Infectious disease underreporting is predicted by country-level preparedness, politics, and pathogen severity.

Meadows et al.

Contents

I. Variable profiles **2**

income 2

STI 3

epidemic 4

Government dissemination of false information abroad 4

Government dissemination of false information domestic 5

Government Internet filtering in practice 5

Government censorship effort — Media 6

Print/broadcast media critical 7

Media bias 7

Public sector corrupt exchanges 8

EPI 9

Year 9

CFR 10

II. GI data exploration **10**

Distribution of EPI 11

Distribution of income 12

Media bias 12

III. Collinearity assessment **12**

V-DEM variable colinearity 13

EPI vs. V-DEM variables 13

EPI vs. income 16

IV. Model selection **17**

All subsets regression 17

ANOVA model comparison 18

V. Model results **18**
Top model 18
Parsimonious model 1 19
Parsimonious model 2 19
Model diagnostics 20

I. Variable profiles

This section contains:

- A description of each variable. For Varieties of Democracy (V-Dem) variables, printing the information returned from the `var_info()` function from the `vdemdata` package. `var_info()` prints to the console basic information on a specific variable as given in the codebook of the V-Dem dataset.
- Results of univariate models for each predictor variable. Results are from a binomial model without robust standard errors.
- A plot showing the relationship of the variable with reporting rates collected in the literature search.
- A plot showing the distribution of values in the data set.

income

Column name in data: income

Description: World Bank income classification.

N: 112

term	estimate	p.value
(Intercept)	-0.004333	0.9965
incomeLower middle income	-2.289	0.05684
incomeUpper middle income	-1.223	0.2938
incomeHigh income	-0.08218	0.9364

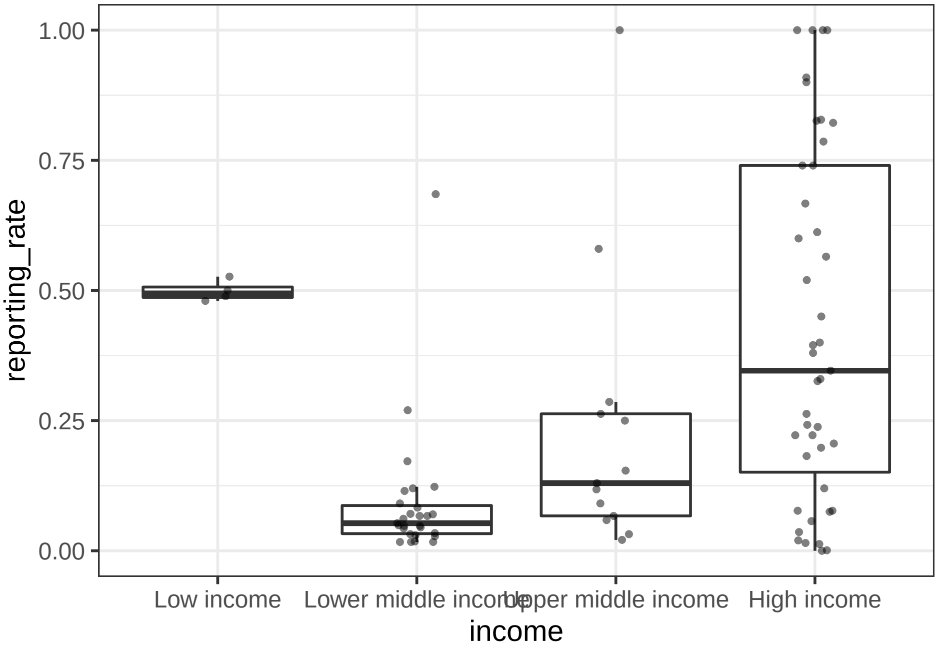

STI

Column name in data: STI

N: 112

term	estimate	p.value
**(Intercept)**	**-0.8288**	**0.0001489**
**STIYes**	**1.678**	**0.009128**

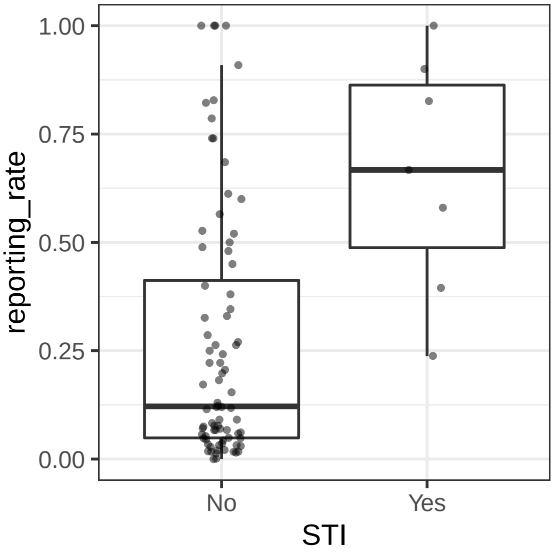

epidemic

Column name in data: epidemic

Description: Was the study estimating reporting rates during an epidemic?

N: 112

term	estimate	p.value
**(Intercept)**	**-0.5639**	**0.01144**
epidemicYes	-0.2563	0.5994

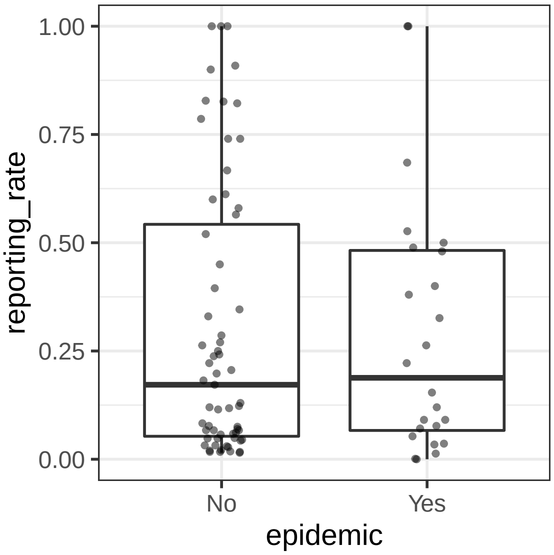

Government dissemination of false information abroad

Column name in data: v2smgovab_ord

Question: How often do the government and its agents use social media to disseminate misleading viewpoints or false information to influence citizens of other countries abroad?

Responses: 0: Extremely often. The government disseminates false information on all key political issues. 1: Often. The government disseminates false information on many key political issues. 2: About half the time. The government disseminates false information on some key political issues, but not others. 3: Rarely. The government disseminates false information on only a few key political issues. 4: Never, or almost never. The government never disseminates false information on key political issues.

N: 87

term	estimate	p.value
**(Intercept)**	**-3.004**	**0.002631**
**v2smgovab_ord**	**0.7036**	**0.02187**

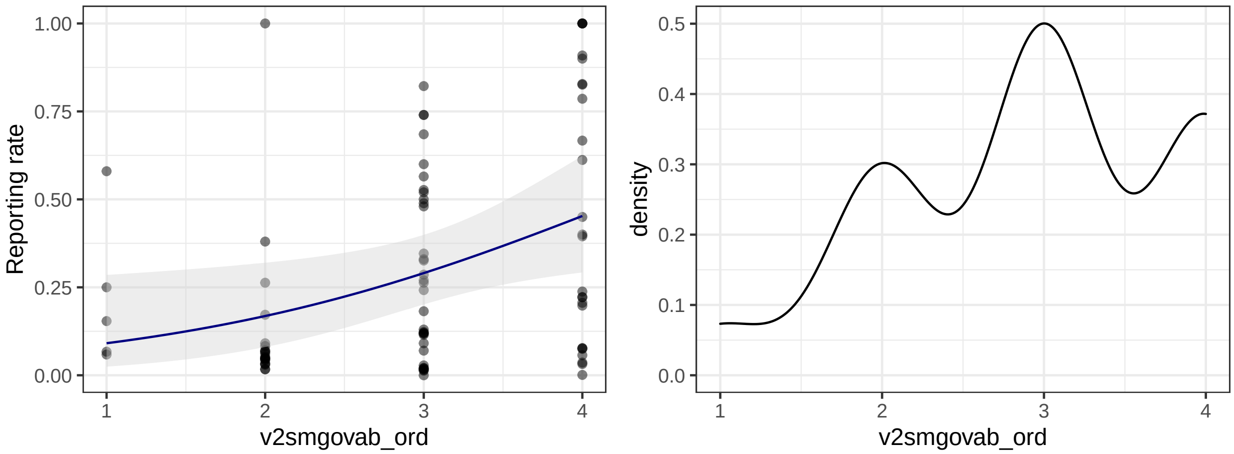

Government dissemination of false information domestic

Column name in data: v2smgovdom_ord

Question: How often do the government and its agents use social media to disseminate misleading viewpoints or false information to influence its own population?

Responses: 0: Extremely often. The government disseminates false information on all key political issues. 1: Often. The government disseminates false information on many key political issues. 2: About half the time. The government disseminates false information on some key political issues, but not others. 3: Rarely. The government disseminates false information on only a few key political issues. 4: Never, or almost never. The government never disseminates false information on key political issues.

N: 87

term	estimate	p.value
**(Intercept)**	**-2.187**	**0.01194**
v2smgovdom_ord	0.4307	0.1018

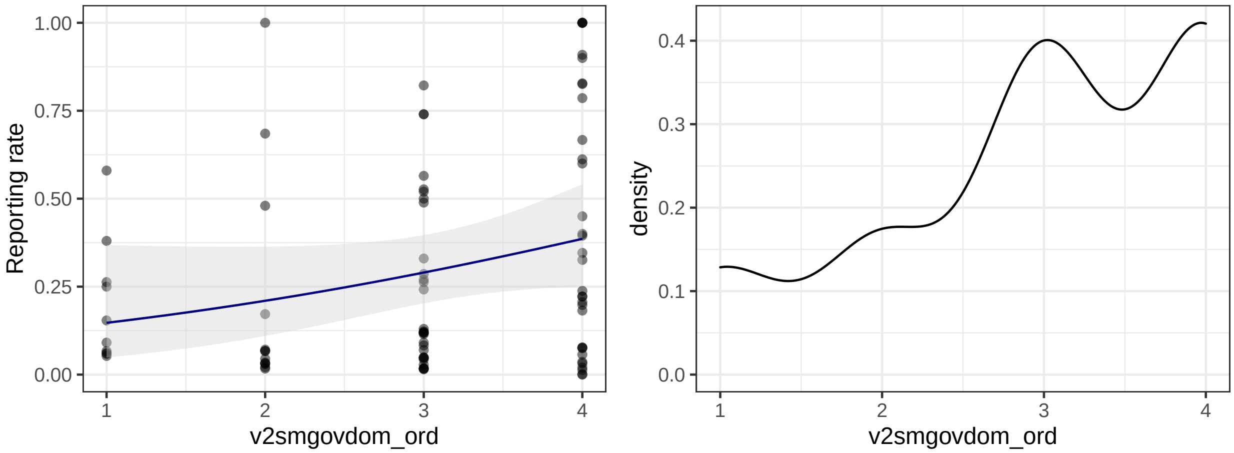

Government Internet filtering in practice

Column name in data: v2smgovfilprc_ord

Question: How frequently does the government censor political information (text, audio, images, or video) on the Internet by filtering (blocking access to certain websites)?

Responses: 0: Extremely often. It is a regular practice for the government to remove political content, except to sites that are pro-government. 1: Often. The government commonly removes online political content, except sites that are pro-government. 2: Sometimes. The government successfully removes about half of the critical online political content. 3: Rarely. There have been only a few occasions on which the government removed political content. 4: Never, or almost never. The government allows Internet access that is unrestricted, with the exceptions mentioned in the clarifications section.

N: 87

term	estimate	p.value
**(Intercept)**	**-2.622**	**0.003538**
**v2smgovfilprc__ord**	**0.5693**	**0.03411**

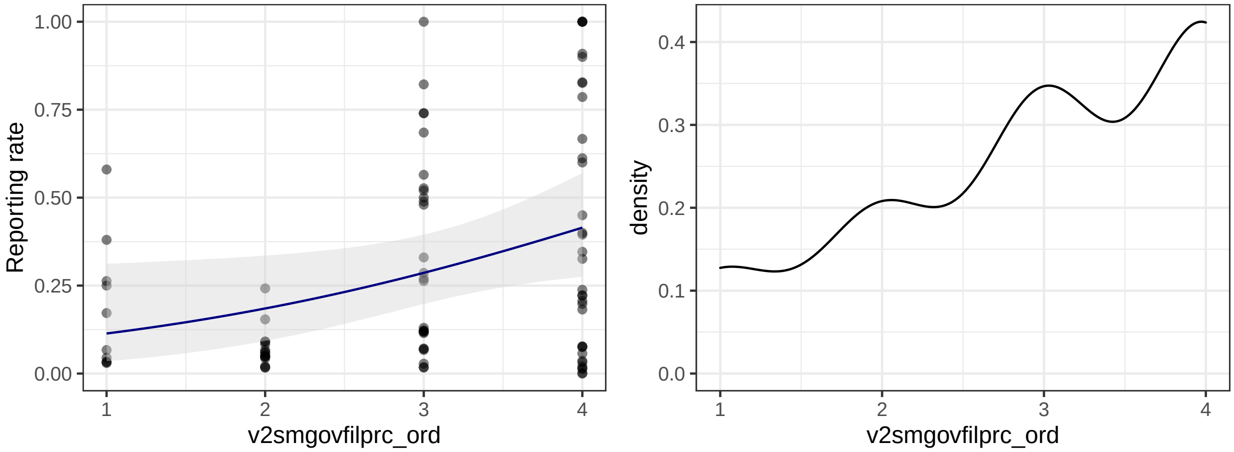

Government censorship effort — Media

Column name in data: v2mecenefm__ord

Question: Does the government directly or indirectly attempt to censor the print or broadcast media?

Responses: 0: Attempts to censor are direct and routine.1: Attempts to censor are indirect but nevertheless routine.2: Attempts to censor are direct but limited to especially sensitive issues.3: Attempts to censor are indirect and limited to especially sensitive issues.4: The government rarely attempts to censor major media in any way, and when such exceptional attempts are discovered, the responsible officials are usually punished.

N: 112

term	estimate	p.value
**(Intercept)**	**-2.049**	**0.005081**
**v2mecenefm__ord**	**0.4378**	**0.03527**

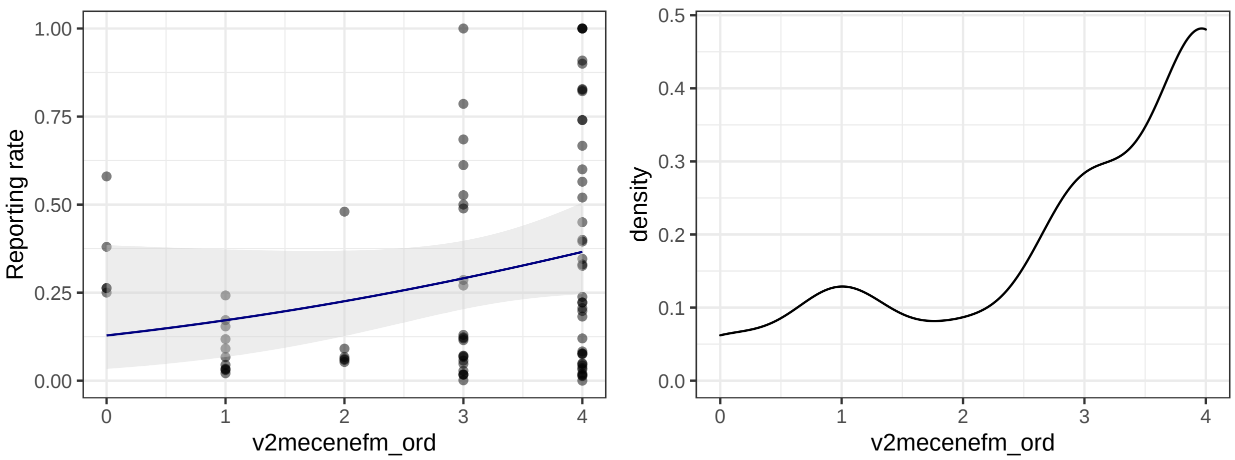

Print/broadcast media critical

Column name in data: v2mecrit_ord

Question: Of the major print and broadcast outlets, how many routinely criticize the government?

Responses: 0: None.1: Only a few marginal outlets.2: Some important outlets routinely criticize the government but there are other important outlets that never do.3: All major media outlets criticize the government at least occasionally.

N: 112

term	estimate	p.value
**(Intercept)**	**-1.949**	**0.02182**
v2mecrit_ord	0.5017	0.0989

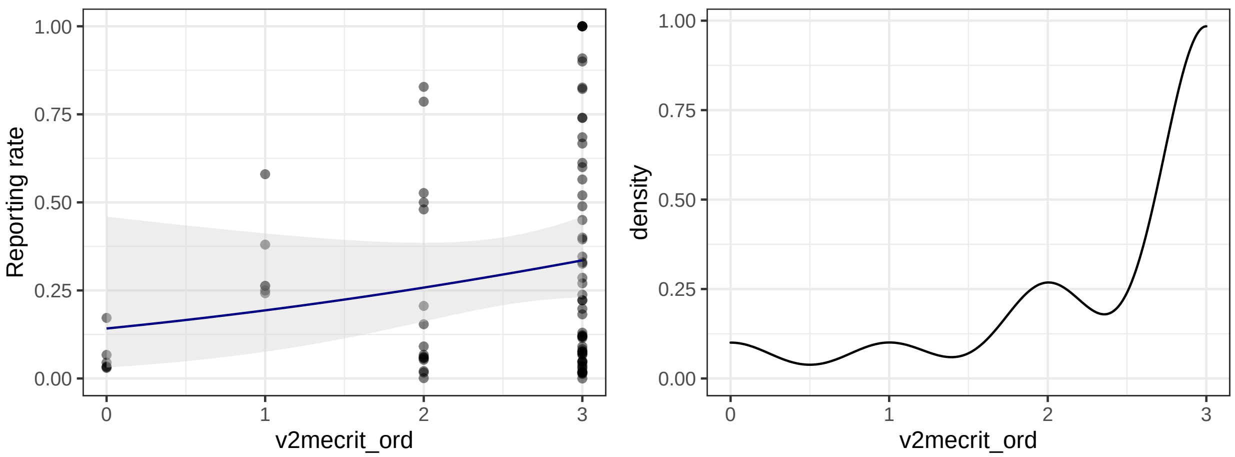

Media bias

Column name in data: v2mebias_ord

Question: Is there media bias against opposition parties or candidates?

Responses: 0: The print and broadcast media cover only the official party or candidates, or have no political coverage, or there are no opposition parties or candidates to cover.1: The print and broadcast media cover more than just the official party or candidates but all the opposition parties or candidates receive only negative coverage. 2: The print and broadcast media cover some opposition parties or candidates more or less impartially, but they give only negative or no coverage to at least one newsworthy party or candidate.3: The print and broadcast media cover opposition parties or candidates more or less impartially, but they give an exaggerated *amount* of coverage to the governing party or candidates.4: The print and broadcast media cover all newsworthy parties and candidates more or less impartially and in proportion to their newsworthiness.

N: 112

term	estimate	p.value
**(Intercept)**	**-2.127**	**0.01169**
v2mebias_ord	0.4344	0.05705

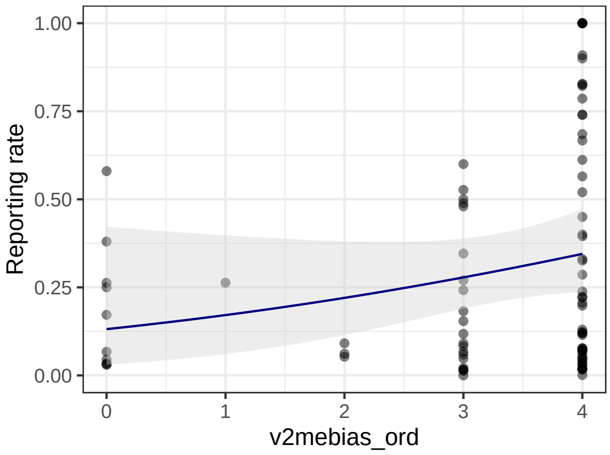

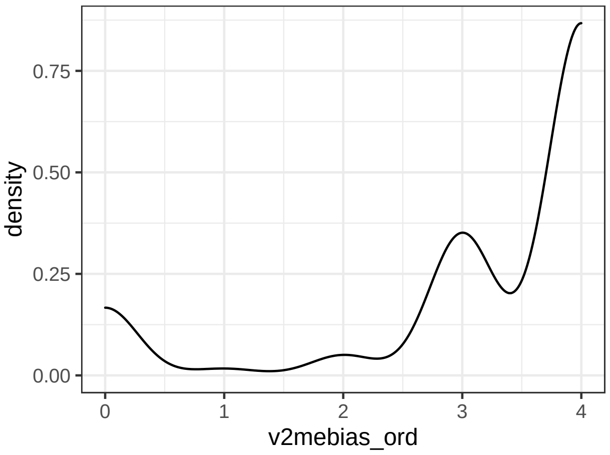

Public sector corrupt exchanges

Column name in data: v2excrptps_ord

Question: How routinely do public sector employees grant favors in exchange for bribes, kickbacks, or other material inducements?

Responses: 0: Extremely common. Most public sector employees are systematically involved in petty but corrupt exchanges almost all the time.1: Common. Such petty but corrupt exchanges occur regularly involving a majority of public employees.2: Sometimes. About half or less than half of public sector employees engage in such exchanges for petty gains at times.3: Scattered. A small minority of public sector employees engage in petty corruption from time to time.4: No. Never, or hardly ever.

N: 112

term	estimate	p.value
**(Intercept)**	**-2.051**	**0.0007245**
**v2excrptps_ord**	**0.6151**	**0.008966**

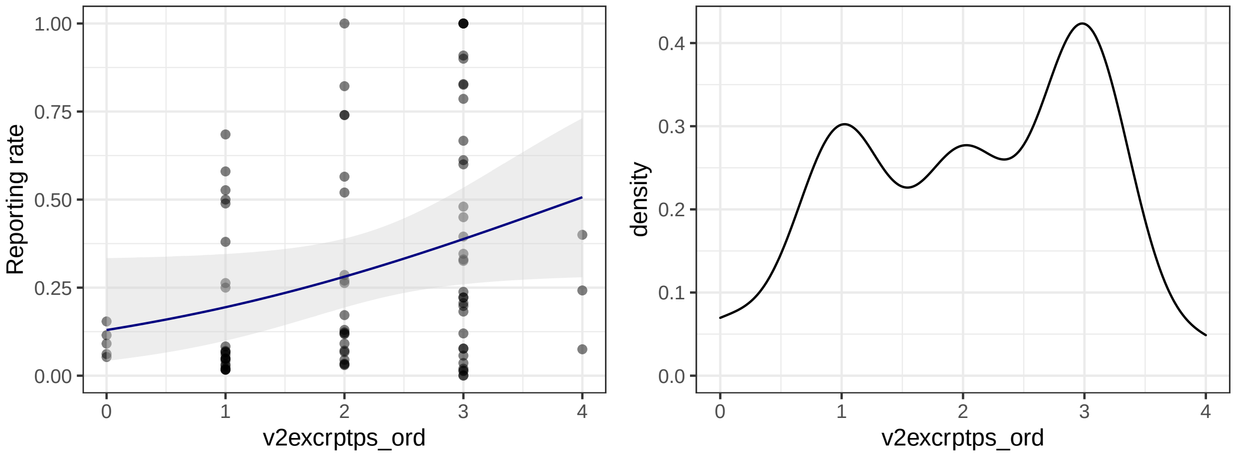

EPI

Column name in data: EPI

Description: Epidemic preparedness index (0 = lowest possible preparedness score; 100 = highest possible preparedness score).

N: 112

term	estimate	p.value
**(Intercept)**	**-4.148**	**0.006459**
**EPI**	**0.04429**	**0.01764**

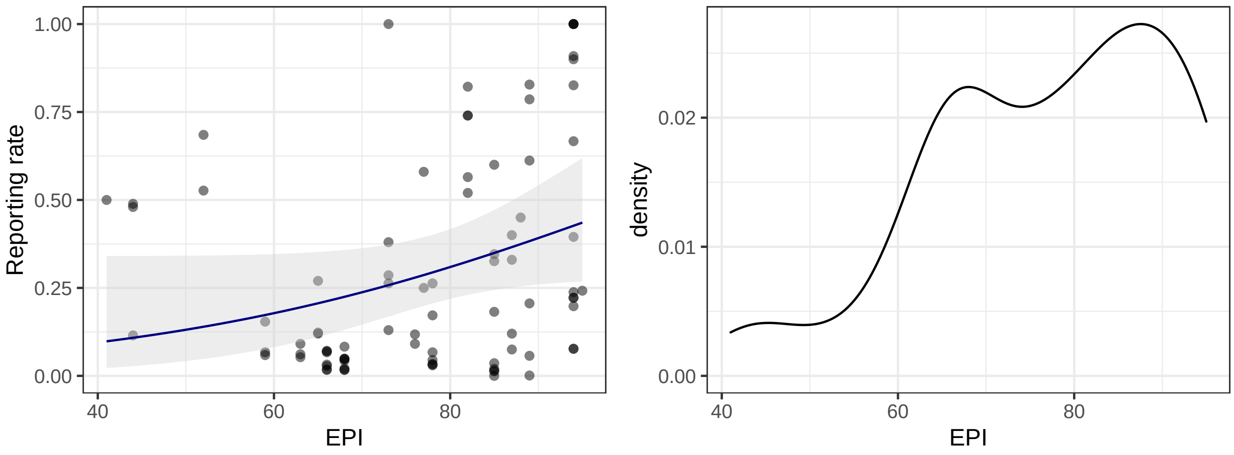

Year

Column name in data: year

Description: The year the study began.

N: 112

term	estimate	p.value
(Intercept)	55.63	0.1665
year	-0.02809	0.1619

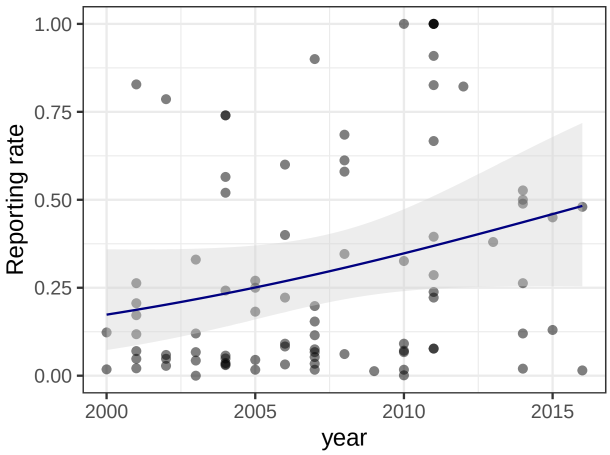

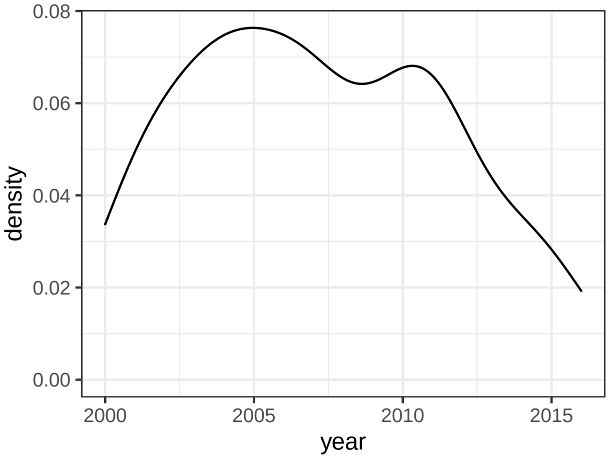

CFR

Column name in data: CFR

Description: Case fatality rate.

N: 112

term	estimate	p.value
**(Intercept)**	**-0.9109**	**8.408e-05**
**CFR**	**0.0377**	**0.009574**

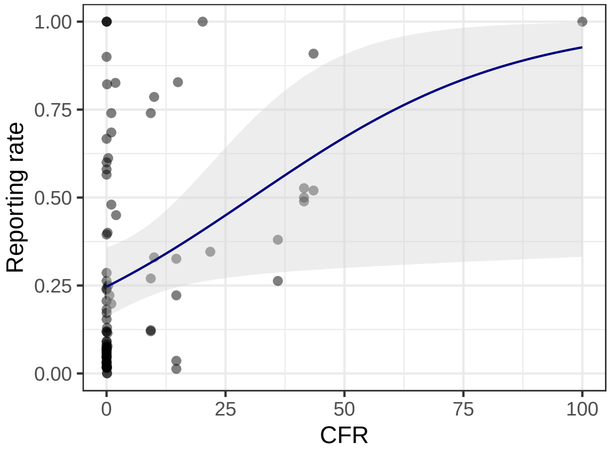

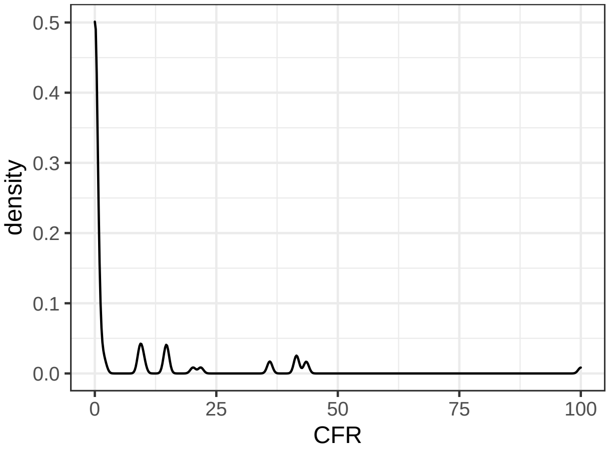

II. GI data exploration

Salmonella and Campylobacter reporting rates (GI data) were excluded from the main analysis owing to the bias in these data towards high income, European countries.

Distribution of EPI

This figure shows the distribution of EPI scores in the GI data (red) versus the reporting rate data used in the analysis (blue). A Kolmogorov-Smirnov test shows a significant difference in the distribution of EPI from these two datasets ($p < 0.001$).

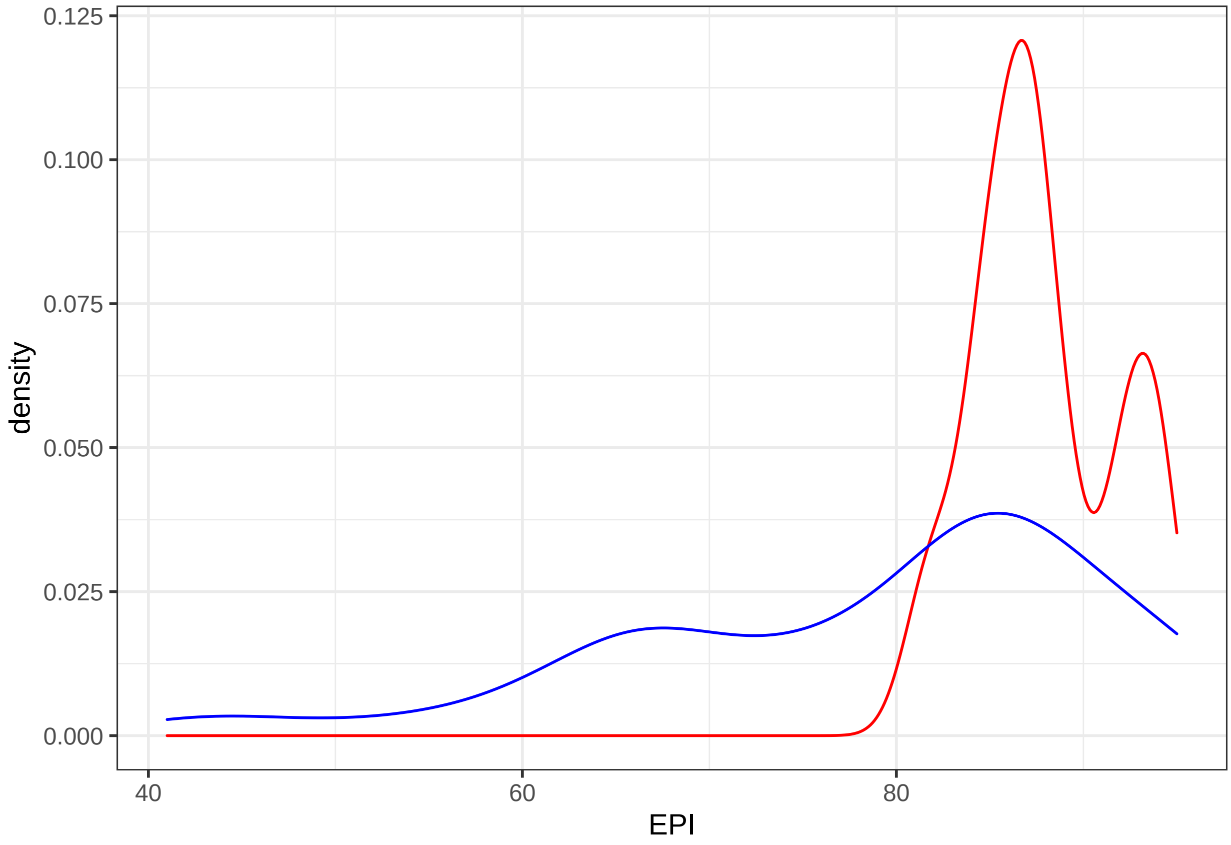


```
##
##  Two-sample Kolmogorov-Smirnov test
##
## data:  giDat$EPI and reportingDat$EPI
## D = 0.4375, p-value = 2.55e-10
## alternative hypothesis: two-sided
```

EPI summary from GI data :

```
##    Min. 1st Qu.  Median    Mean 3rd Qu.    Max.
##   81.00   85.00   87.00   87.88   92.00   94.00
```

EPI summary from analysis data :

```
##    Min. 1st Qu.  Median    Mean 3rd Qu.    Max.
##   41.00   68.00   85.00   78.51   87.00   95.00
```

Distribution of income

Observations by income group in GI data:

```
##                income observations
## 1:         High income          121
## 2: Upper middle income            6
```

Observations by income group analysis data:

```
##                income Observations
## 1: Upper middle income           16
## 2:         High income           65
## 3:          Low income            4
## 4: Lower middle income           27
```

Media bias

Observations by media bias score gi data:

```
##    v2mebias_ord Observations
## 1:            4          110
## 2:            3           16
## 3:            2            1
```

Observations by media bias score analysis data:

```
##    v2mebias_ord Observations
## 1:            4           75
## 2:            0           10
## 3:            3           23
## 4:            2            3
## 5:            1            1
```

III. Collinearity assessment

Assessing correlation among significant univariate variables before entering variables into global model

V-DEM variable colinearity

Take away: A lot of collinearity among V-Dem variables; will consider P-value of univarite models and correlation with EPI when deciding which variables to remove.

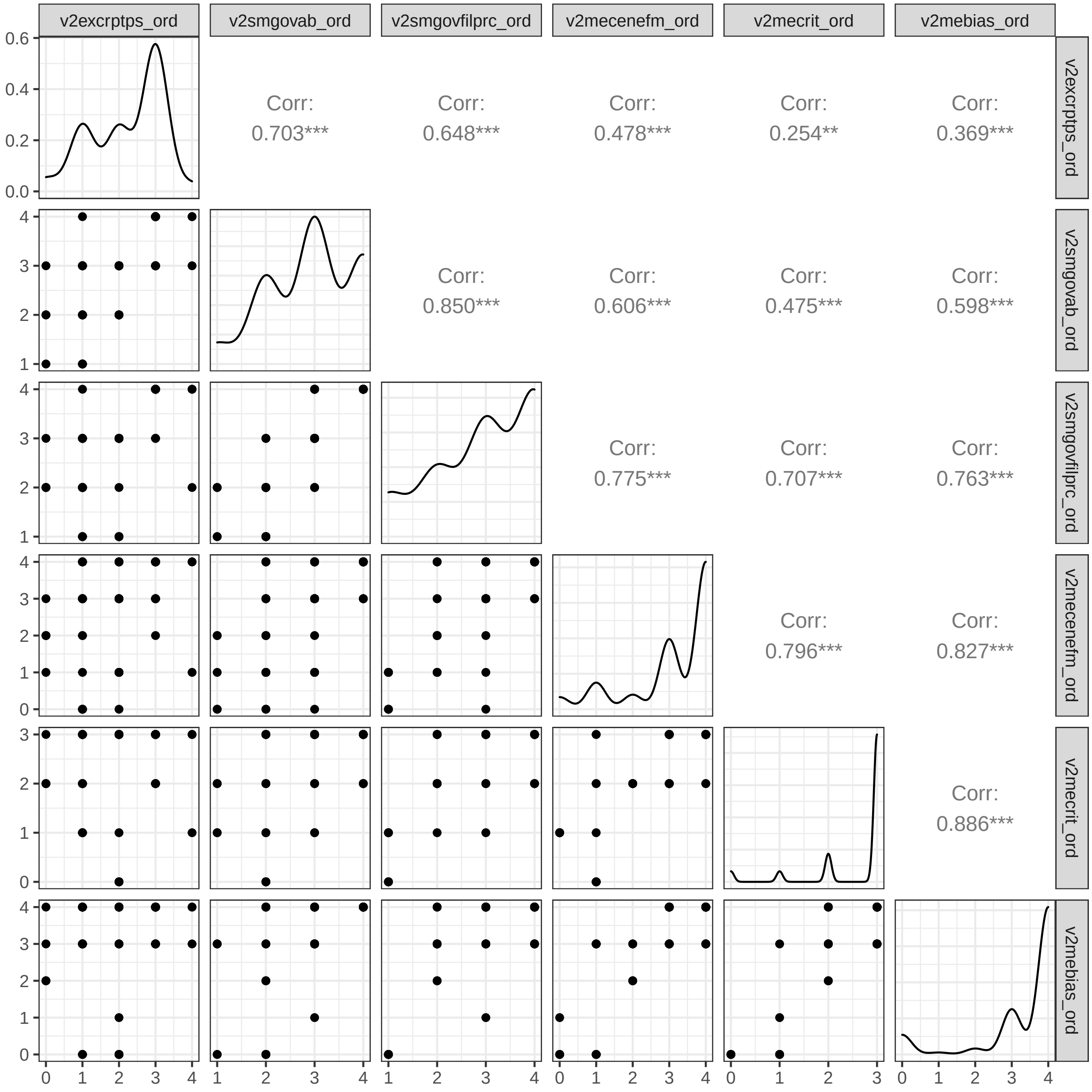

EPI vs. V-DEM variables

- Plots of each V-DEM variable (y-axis) considered against EPI (x-axis)
- Main take aways:
  - Corruption-related variables are strongly negatively correlated with EPI. Not surprising, since it is a component of EPI

– Print/broadcast media variables are not highly correlated with EPI. Can provide additional information to the model.
– Coordinated Information Operations variables (online media, internet censorship) are mildly correlatd with EPI

v2excrptps_ord

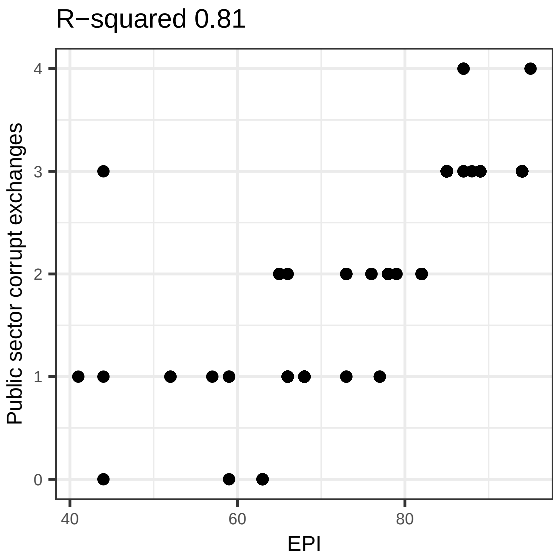

v2smgovab_ord

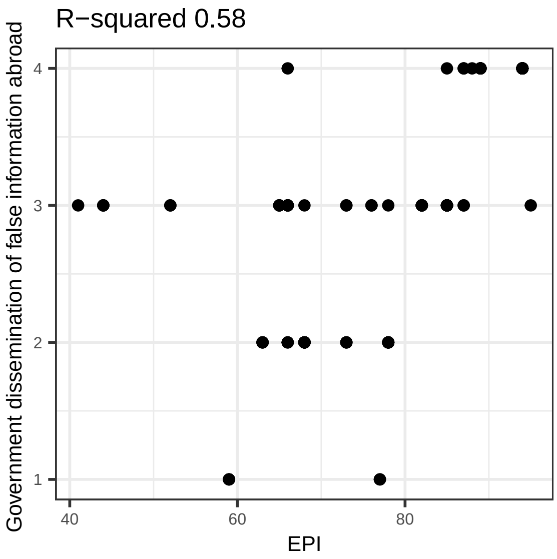

v2smgovfilprc_ord

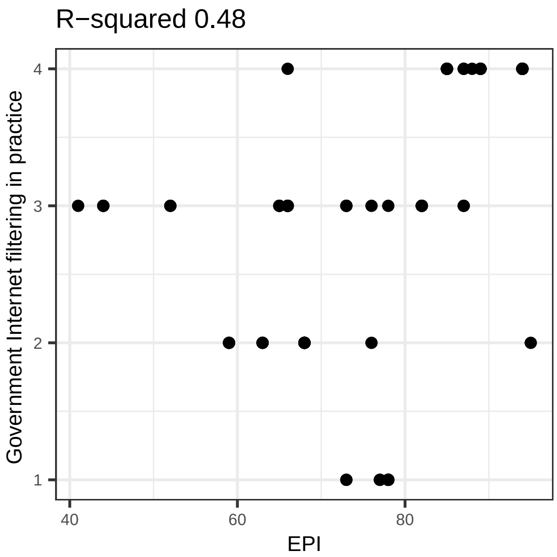

v2mecenefm_ord

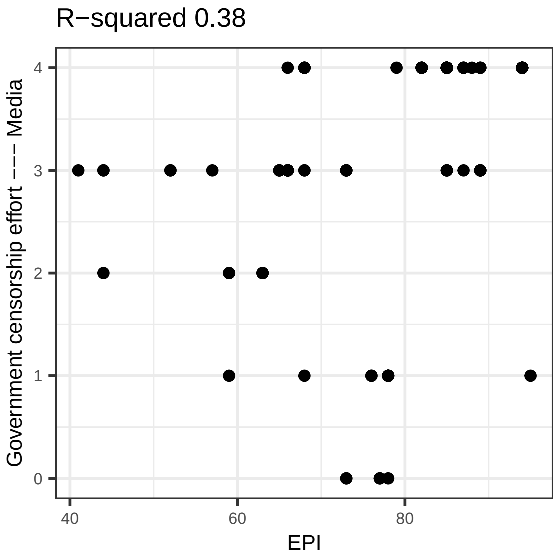

v2mecrit_ord

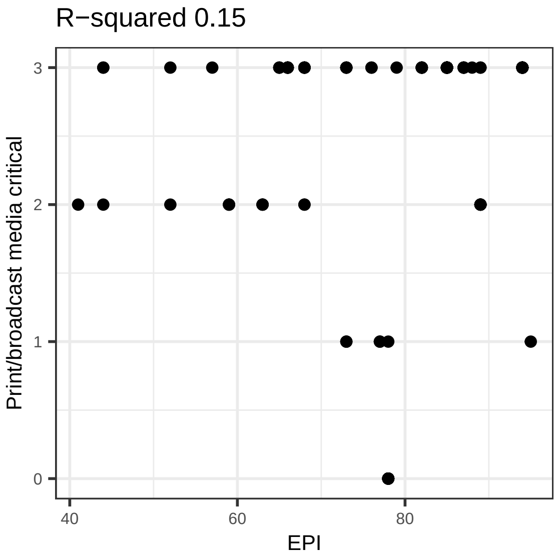

v2mebias_ord

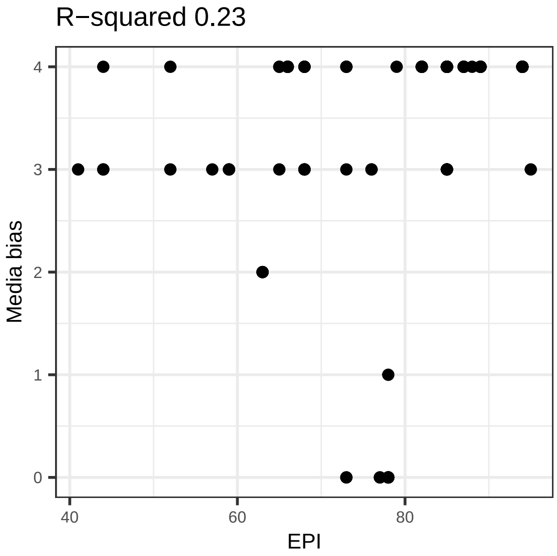

EPI vs. income

- Take away: EPI and income highly correlated.
    - Income is also a component of EPI

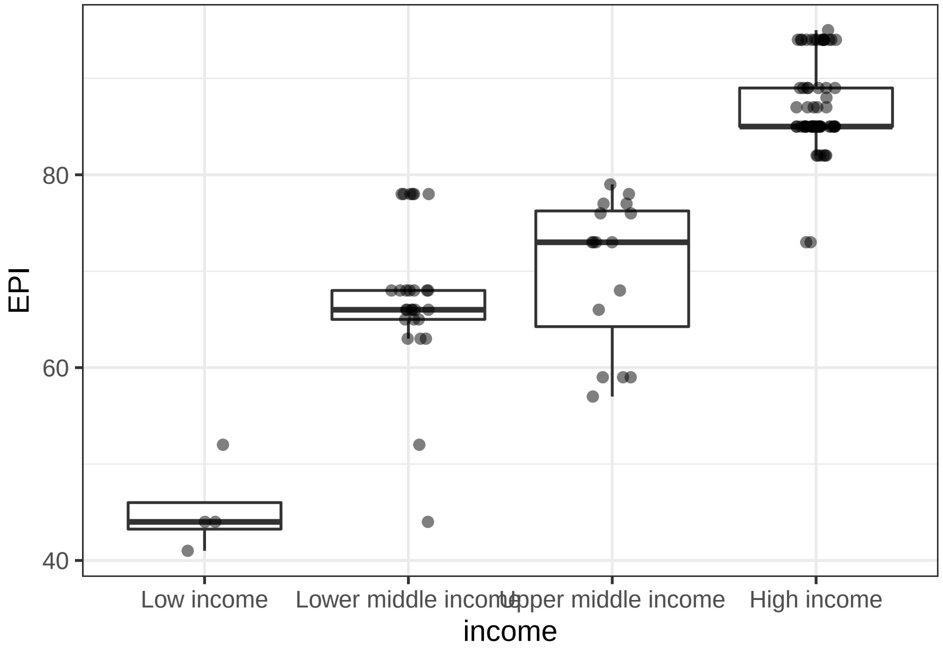

IV. Model selection

Global model variables:

- EPI
- v2mebias_ord
- STI
- CFR

Excluded due to collinearity:

- Income group: highly correlated with EPI
- v2smgovfilprc_ord (Government Internet filtering in practice): highly correlated with v2smgovab_ord and moderately correlated with EPI. Additionally, fewer data points available for model fitting due to limited years of data collection.
- v2smgovdom_ord (Government dissemination of false information domestically): highly correlated with v2smgovab_ord and v2smgovfilprc_ord. Additionally, fewer data points available for model fitting due to limited years of data collection.
- v2mecenefm_ord (Government censorship effort — media): correlated with v2mebias_ord and EPI.

All subsets regression

- Showing the models with ΔAIC less than 2 from all subsets regression.

(Intercept)	CFR	EPI	STI	v2mebias_ord	df	logLik	AICc	delta	weight
-4.078	0.04736	0.02378	+	0.2705	5	-38.57	87.88	0	0.3753
-2.423	0.04359	NA	+	0.3274	4	-39.73	87.95	0.07527	0.3615

(Intercept)	CFR	EPI	STI	v2mebias_ord	df	logLik	AICc	delta	weight
-3.609	0.04515	0.02935	+	NA	4	-40.05	88.59	0.7095	0.2632

ANOVA model comparison

- If ANOVA comparison is significant, this supports inclusion of the additional variable over the more parsimonious model.

Top model vs. parsimonious model 1:

```
## Robust Wald Test Table
##
## Model 1: reporting_rate ~ STI + CFR + EPI + v2mebias_ord
## Model 2: reporting_rate ~ STI + CFR + v2mebias_ord
## Models fitted by method 'BY'
##
##   pseudoDf Test.Stat Df Pr(>chisq)
## 1       -5
## 2       83   -3.6821 -1      0.055 .
## ---
## Signif. codes:  0 '***' 0.001 '**' 0.01 '*' 0.05 '.' 0.1 ' ' 1
```

Top model vs. parsimonious model 2:

```
## Robust Wald Test Table
##
## Model 1: reporting_rate ~ STI + CFR + EPI + v2mebias_ord
## Model 2: reporting_rate ~ STI + CFR + EPI
## Models fitted by method 'BY'
##
##   pseudoDf Test.Stat Df Pr(>chisq)
## 1       -5
## 2       83    -3.986 -1    0.04588 *
## ---
## Signif. codes:  0 '***' 0.001 '**' 0.01 '*' 0.05 '.' 0.1 ' ' 1
```

V. Model results

- Using robust regression to account for nonconstant variance.
- Terms with P<0.05 are in **bold**.
- Including variable importance to indicate which variables have the largest effect on reporting rate.
- Including the top 3 models for comparison.

Top model

term	estimate	std.error	statistic	p.value
**(Intercept)**	**-6.198**	**1.51**	**-4.106**	**4.03e-05**

term	estimate	std.error	statistic	p.value
**STIYes**	**2.487**	**0.5664**	**4.392**	**1.125e-05**
**CFR**	**0.07016**	**0.01403**	**5.001**	**5.703e-07**
**EPI**	**0.03631**	**0.01571**	**2.311**	**0.02082**
**v2mebias_ord**	**0.4508**	**0.1978**	**2.279**	**0.02269**

Variable importance

- Comparing reduction in deviance when each term is entered into the model last.

Variable	Reduction in deviance
CFR	10.56
STI	6.553
EPI	1.721
v2mebias_ord	2.154

Parsimonious model 1

- The model with the second lowest AICc has one fewer term.

term	estimate	std.error	statistic	p.value
**(Intercept)**	**-3.74**	**0.91**	**-4.11**	**3.961e-05**
**STIYes**	**2.816**	**0.5758**	**4.891**	**1.003e-06**
**CFR**	**0.06782**	**0.01154**	**5.875**	**4.238e-09**
**v2mebias_ord**	**0.5637**	**0.2301**	**2.45**	**0.01429**

Parsimonious model 2

- The model with the third lowest AICc also has one fewer term.

term	estimate	std.error	statistic	p.value
**(Intercept)**	**-5.407**	**1.427**	**-3.789**	**0.0001513**
**STIYes**	**2.419**	**0.4415**	**5.479**	**4.278e-08**
**CFR**	**0.06754**	**0.01171**	**5.766**	**8.133e-09**
**EPI**	**0.04643**	**0.01646**	**2.821**	**0.004793**

Model diagnostics

- From left to right: residual vs. fitted, actual vs. fitted (transformed to response scale), distribution of residuals.
- These are from non-robust model.

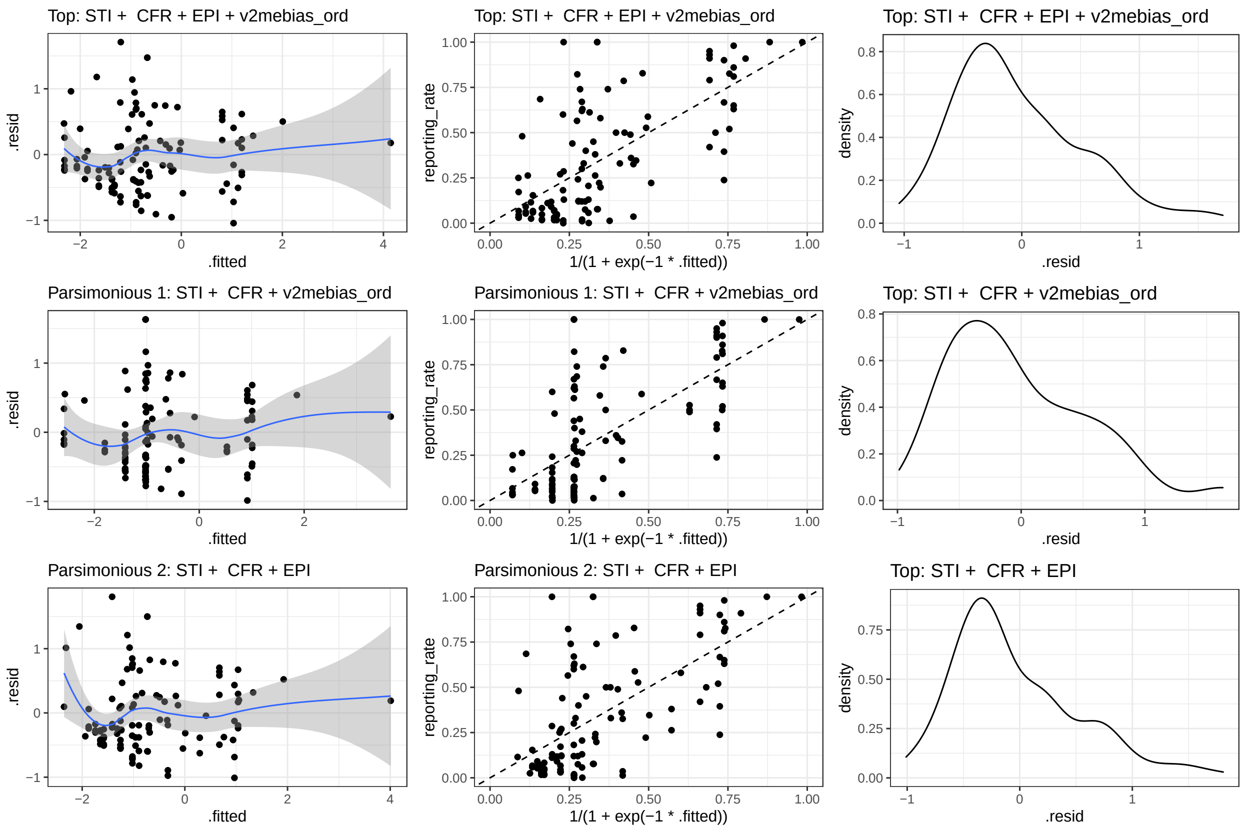

Supplement: Supplemental data [file Supp_DataS1.pdf]
